# Human Umbilical Cord Mesenchymal Stem Cells Ameliorate Diabetic Neuropathic Pain via TRPV1-[Ca^2+^]i-AMPK Signaling-Mediated Mitochondrial Restoration in Schwann Cells

**DOI:** 10.1155/sci/5533136

**Published:** 2025-11-26

**Authors:** Yi-kun Zhou, Jun-dong He, Xiao-chun Yang, Li-fen Yang, Pei-yu Jiang, Zhi Liang, Yang Ou

**Affiliations:** ^1^Department of Endocrinology and Metabolism, First People's Hospital of Yunnan Province, The Affiliated Hospital of Kunming University of Science and Technology, Kunming 650032, Yunnan, China; ^2^Medical School, Kunming University of Science and Technology, Kunming, Yunnan, China; ^3^Department of Ophthalmology, First People's Hospital of Yunnan Province, The Affiliated Hospital of Kunming University of Science and Technology, Kunming, Yunnan, China; ^4^Department of Information Center, First People's Hospital of Yunnan Province, The Affiliated Hospital of Kunming University of Science and Technology, Kunming 650032, Yunnan, China

**Keywords:** AMPK, diabetic peripheral neuropathy, human umbilical cord mesenchymal stem cells, intracellular calcium, mitochondrial dynamics, TRPV1

## Abstract

**Background:**

The therapeutic potential of human umbilical cord mesenchymal stem cells (HUCMSCs) for diabetic peripheral neuropathy (DPN) and the underlying mechanisms involving transient receptor potential vanilloid 1 (TRPV1) signaling remain incompletely defined.

**Objective:**

This study aimed to elucidate the role of the TRPV1-[Ca^2+^]i-AMPK signaling axis in mediating the beneficial effects of HUCMSCs on neuropathic pain and Schwann cell (SC) dysfunction in DPN.

**Methods:**

A murine model of DPN was established. Mechanical allodynia and thermal hyperalgesia were assessed using Von Frey filaments and the KW-LB hot plate test, respectively. Primary mouse SCs were isolated and cultured under high glucose (HG) conditions. Intracellular calcium ([Ca^2+^]i) levels were quantified by flow cytometry. Protein expression (TRPV1, p-TRPV1, AMPK, p-AMPK, cleaved-caspase-3, Bax, Bcl-2, Drp1, PGC-1α, TFAM, Mfn2) was analyzed via Western blotting. Apoptosis and cell proliferation were evaluated using TUNEL staining and the CCK-8 assay, respectively. Specific inhibitors (AMG9810 for TRPV1 and compound C for AMPK) were employed to probe pathway involvement.

**Results:**

HUCMSC administration significantly alleviated mechanical allodynia and thermal hyperalgesia in diabetic mice. In vitro, HUCMSC coculture counteracted HG-induced effects in SCs by: (1) increasing the p-TRPV1/TRPV1 ratio and [Ca^2+^]i influx (effects blocked by AMG9810); (2) reducing apoptosis (decreased cleaved-caspase-3/Bax, increased Bcl-2); (3) enhancing the p-AMPK/AMPK ratio (attenuated by both AMG9810 and compound C); and (4) promoting mitochondrial homeostasis, increasing PGC-1α, TFAM, and Mfn2 expression, mitochondrial membrane potential and ATP levels, and decreasing Drp1 expression. These mitochondrial improvements were reversed by compound C.

**Conclusion:**

HUCMSCs ameliorate diabetic neuropathic pain primarily through activation of the TRPV1-[Ca^2+^]i-AMPK signaling pathway in SCs, which may provide a new molecular target for enhancing the clinical therapeutic effect of HUCMSCs on DPN.

## 1. Introduction

The global diabetes pandemic is projected to affect 642 million individuals by 2040 [1], with diabetic peripheral neuropathy (DPN) emerging as its most prevalent and debilitating complication. Despite hyperglycemia being the primary driver of neural damage [2], current treatments fail to halt disease progression, highlighting an urgent need for novel therapies.

Mesenchymal stem cells (MSCs) offer promising regenerative potential for DPN. Studies have shown that human umbilical cord MSCs (HUCMSCs) can improve the nerve conduction velocity in DPN patients [3]. HUCMSCs treatment can significantly improve the diabetic-induced femoral nerve neuropathy in rats [4]. Furthermore, our earlier research also demonstrated that HUCMSCs can significantly reduce the damage to DPN in mice [5]. In DNP, the transient receptor potential vanilloid type 1 (TRPV1) can participate in pain regulation through multiple pathways [6–8]. However, it remains to be investigated whether HUCMSCs can affect TRPV1 to improve DNP. Critically, Schwann cells (SCs), the guardians of peripheral nerve integrity [9–11], undergo apoptosis and mitochondrial dysfunction in DPN [12], yet their role in MSC-mediated recovery remains unexplored.

While TRPV1's function in sensory neurons is well-established [13–17], its impact on SCs, particularly in modulating calcium flux ([Ca^2+^]i), AMPK signaling, and mitochondrial dynamics, is entirely unknown. This gap is pivotal given that TRPV1 activation rescues neuronal damage in diabetes [18, 19]; TRPV1-[Ca^2+^]i-AMPK crosstalk coordinates mitochondrial biogenesis/fission in other cell types [20–24]; and mitochondrial fragmentation drives SC apoptosis in DPN [25–27].

SCs play a crucial role in the peripheral nervous system by providing myelination, maintaining, nourishing, and repairing axons and neurons, as well as producing neurotrophic factors. Here, we consider SCs as the important target of HUCMSC therapy and elucidate a novel TRPV1-[Ca^2+^]i-AMPK axis that restores mitochondrial homeostasis. This work bridges three critical domains: MSC therapy, TRPV1 signaling in non-neuronal cells, and mitochondrial pathophysiology in DPN, offering a unified mechanism for neuroprotection.

## 2. Materials and Methods

### 2.1. Cell Culture and Treatment

HUCMSCs were obtained from the Cell Therapy Center of Kunming Yan'an Hospital. These cells were cultured in DMEM low-glucose medium (Hyclone, USA) supplemented with 10% fetal bovine serum (FBS, Gibco, Australia). Primary mouse SCs were isolated and cultured in DMEM/F12 medium (Hyclone, USA) supplemented with 10% FBS and 1% penicillin–streptomycin (Hyclone, USA). All cells were maintained in a humidified incubator at 37°C with 5% CO_2_ and 95% humidity.

### 2.2. Primary Culture of Mouse SCs

The T25 culture flasks were coated with 200 μg/mL poly-L-lysine. Mouse sciatic nerves were dissected, the epineurium removed, and the tissue minced. Type I collagenase was used for digestion for 2 h, followed by termination with a small amount of serum. The cells were centrifuged at 1500 rpm for 5 min, washed once with PBS, and resuspended in DMEM/F12 medium containing 10% FBS. The cell suspension was then transferred to prepared culture flasks and maintained at 37°C with 5% CO_2_. For high glucose (HG) modeling, cells were treated with 25 mM glucose for 24 h. After HG treatment, the medium was replaced with normal medium, and 10 μM AMG9810 (AMG9810) is an antagonist of TRPV1. It has the ability to selectively and competitively block the activation of TRPV1 receptors, or compound C (an AMPK inhibitor that can prevent the phosphorylation of AMPK, thus inhibiting its activity) was added. Following a 2-h incubation at 37°C, stem cells were added for coculture for 24 h. The culture medium and SCs were then collected for further analysis.

### 2.3. Animal Model Establishment

C57BL/6 mice are widely used in diabetes research due to their susceptibility to streptozotocin (STZ)-induced diabetes. After balancing statistical requirements, ethical responsibilities, and resource constraints, 50 male C57BL/6 mice (4–6 weeks old, 18–20 g) were selected and purchased from the Animal Experiment Center of Kunming Medical University. The mice were randomly divided into 5 groups (*n* = 10/group) using a computer-generated randomization list, including a normal control group (normal), a DPN model group, a stem cell therapy group (HUCMSC), and intervention groups (e.g., TRPV1 or AMPK inhibitor groups). The entire experiment followed a double-blind design, with researchers unaware of group allocations during data analysis. All mice were housed under identical standardized conditions, with feeding and standardized procedures performed by the same researcher. Mice were housed under controlled conditions (22 ± 2°C, 12-h light/dark cycle) with free access to food and water. The normal control group did not undergo diabetes induction, while the other groups received STZ to establish the diabetic model. STZ selectively destroys pancreatic β-cells, mimicking type 1 diabetes. After 1 week of acclimatization, diabetes was induced by intraperitoneal (IP) injection of STZ at a dose of 50 mg/kg. A second injection was administered 24 h later using the same dose and method. Three days after the second injection, all mice were fasted for 3 h but allowed free access to water. Body weight was measured, and fasting blood glucose levels were determined from tail blood samples. Mice with blood glucose levels ≥16.8 mmol/L were considered successful diabetic models; those failing to meet this criterion (~5%) were excluded from the study. Within 8 weeks postmodeling, mechanical allodynia was assessed weekly using von Frey filaments and thermal hyperalgesia using a KW-LB hot plate analgesia meter. Subsequently, umbilical cord MSCs (2 × 10^4^ cells) were injected into the tail vein every other week for three injections, followed by monitoring changes in neuropathy indicators in DPN mice. The work has been reported in line with the ARRIVE guidelines 2.0.

### 2.4. Von Frey Test

Mechanical allodynia was assessed using calibrated von Frey filaments (BME-404, Institute of Biomedical Sciences, Chinese Academy of Medical Sciences). Prior to the experiment, mice were placed in boxes with glass-covered walls and tops and metal mesh floors. After a 30-min adaptation period, the plantar surface of the hind paw was stimulated with a 0.5 g von Frey filament for 5–10 s per application. If the mouse exhibited paw withdrawal or licking, it was considered a positive response; otherwise, it was negative. The interstimulus interval was 30 s, and each filament was tested five times consecutively. The minimum stimulus intensity that elicited three positive responses was recorded as the mechanical withdrawal threshold (MWT).

### 2.5. Hot Plate Test

Mice were placed in transparent acrylic boxes on a KW-LB hot plate analgesia meter and allowed to adapt for 20–30 min. Heat radiation was focused on the top glass surface, which was initially maintained at 30°C. The hot plate apparatus was activated with a heating rate of 1°C/s, and the time from the start of irradiation until the mouse exhibited paw withdrawal or licking was recorded. To prevent burning the plantar skin, the maximum exposure time was set to 20 s. Each measurement was repeated five times with 3-min intervals between trials, and the average value was recorded as the thermal withdrawal latency (TWL).

### 2.6. Western Blot

Total protein was extracted from SCs, and protein concentration was determined using a BCA kit (Sigma–Aldrich, UK). Target proteins were separated on 10% SDS–PAGE gels and electrotransferred onto PVDF membranes, which were then blocked with 5% bovine serum albumin for 1 h. The membranes were incubated overnight at 4°C with primary antibodies including Bax (Abcam, 1:1000), Bcl-2 (Abcam, 1:1000), cleaved-caspase-3 (Cell Signaling Technology, 1:1000), p-TRPV1 (Thermo, 1:1000), TRPV1 (Abcam, 1:1000), p-AMPK (Abcam, 1:2000), AMPK (Abcam, 1:1000), PGC-1α (Abcam, 1:5000), TFAM (GUSABIO, 1:10,000), Mfn2 (Abcam, 1:2000), and Drp1 (Abcam, 1:1000). The next day, membranes were washed with TBST for 30 min and incubated with corresponding secondary antibodies at room temperature for 1–2 h. Enhanced chemiluminescence (ECL) reagents were used for detection and visualization, and Image J software was employed for quantitative analysis of protein bands.

### 2.7. Flow Cytometry for Ca^2+^ Levels

SCs were digested and collected, washed with PBS, centrifuged at 1500 rpm for 5 min, and the supernatant was discarded. Cells were incubated with a specific Ca^2+^ fluorescent indicator at a concentration of 10 μmol/L at 37°C for 1 h under light-protected conditions. After incubation, cells were centrifuged, and excess dye was removed by washing with PBS. Finally, flow cytometry was performed using excitation and emission wavelengths of 488 and 525 nm, respectively, to detect and record the fluorescence signals.

### 2.8. CCK-8 Assay

Cell viability was assessed using a CCK-8 kit (MCE, China). SCs were seeded at a density of 3000 cells per well in 96-well plates and cultured at 37°C. To induce injury, cells were cultured in medium containing 25 mM glucose for 24 h. Following this, the cells were cocultured with HUCMSCs for 24 h. Then, 10 μL of CCK-8 solution was added to each well, and the plates were incubated in the incubator for 2 h. Absorbance was measured at 450 nm using a microplate reader.

### 2.9. TUNEL Staining

TUNEL staining was performed using a TUNEL Apoptosis Detection Kit (Beyotime, Shanghai, China). SCs were seeded on 14 mm coverslips in 24-well plates for growth and treatment. After the treatment period, SCs were incubated with the TUNEL reaction mixture at 37°C for 1 h under light-protected conditions. The cells were then washed three times with PBS, followed by DAPI staining and mounting. Prepared samples were observed under a fluorescence microscope.

### 2.10. Flow Cytometry for Mitochondrial Membrane Potential

The mitochondrial membrane potential was measured using the JC-1 detection kit (C2006, Beyotime, China). The ratio of mitochondrial membrane potential was defined as the fluorescence intensity of red (JC-1 aggregates)/green (JC-1 monomers) and was analyzed by flow cytometry.

### 2.11. ATP Measurement

The ATP level was evaluated using the ATP Assay Kit (A095-1-1, Jiancheng, Nanjing, China). SCs were seeded into 6-well plates for 24 h. After grouping, the cells were treated with the lysis buffer. Then, the working solution was added to the cell supernatant. Finally, the ATP level was detected using a microplate reader, and the ATP level was standardized to the protein content of each sample.

### 2.12. Statistical Analysis

GraphPad Prism 8.0 was used for statistical analysis and graph generation. All experiments were performed in triplicate with three independent replicates. Data are presented as mean ± standard deviation. Unpaired *t*-tests were used to compare differences between two groups, while one-way ANOVA was used for comparisons among multiple groups. The statistical significance level for all tests was set at *p* < 0.05.

### 2.13. Animal Euthanasia Methods

Weekly monitoring of mouse body weight and general health assessments was conducted. Mice exhibiting severe weight loss (≥20% of baseline body weight), inability to access food or water, or signs of severe distress meeting the humane endpoint criteria were euthanized. Administer an IP injection of 20% sodium pentobarbital solution at a dose of 300 mg/kg. Following the injection, confirm the cessation of heartbeat by palpating the thoracic cavity. Check for dilated pupils and absence of light reflex to verify death.

## 3. Results

### 3.1. HUCMSCs Alleviate Diabetic Neuropathy in Mice

**Figure 1:**
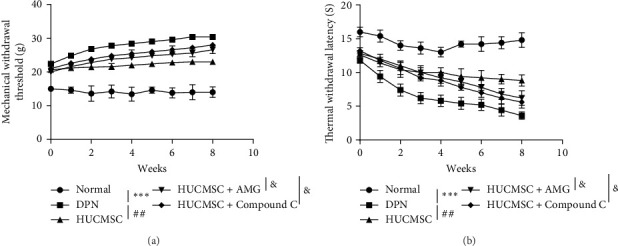
Monitoring of pain and thermal sensation in mice. (A) Stabbing pain sensation and (B) thermal sensation. Compared with the normal group, *⁣*^*∗∗∗*^*p* < 0.001; compared with the DPN group, ^##^*p* < 0.01; and compared with the HUCMSC group, ^&^*p* < 0.05(*n* = 5).

By establishing a mouse model of diabetic neuropathy, we evaluated changes in pain and thermal sensation over an 8-week period. Compared to the control group, DPN mice exhibited significantly reduced mechanical allodynia, as indicated by increased MWTs, and increased thermal hyperalgesia, as shown by shortened thermal withdrawal latencies. Treatment with HUCMSCs improved both mechanical and thermal sensitivity. However, the beneficial effects of HUCMSCs on neural function were attenuated following injection of AMG9810 or compound C (Figure 1A, B). These findings indicate that HUCMSCs can alleviate diabetic neuropathy in mice, and this ameliorative effect is associated with the activation of TRPV1 and AMPK signaling pathways.

### 3.2. HUCMSCs Alleviate HG-Induced SC Injury via the TRPV1-Ca^2+^ Signaling Pathway

**Figure 2:**
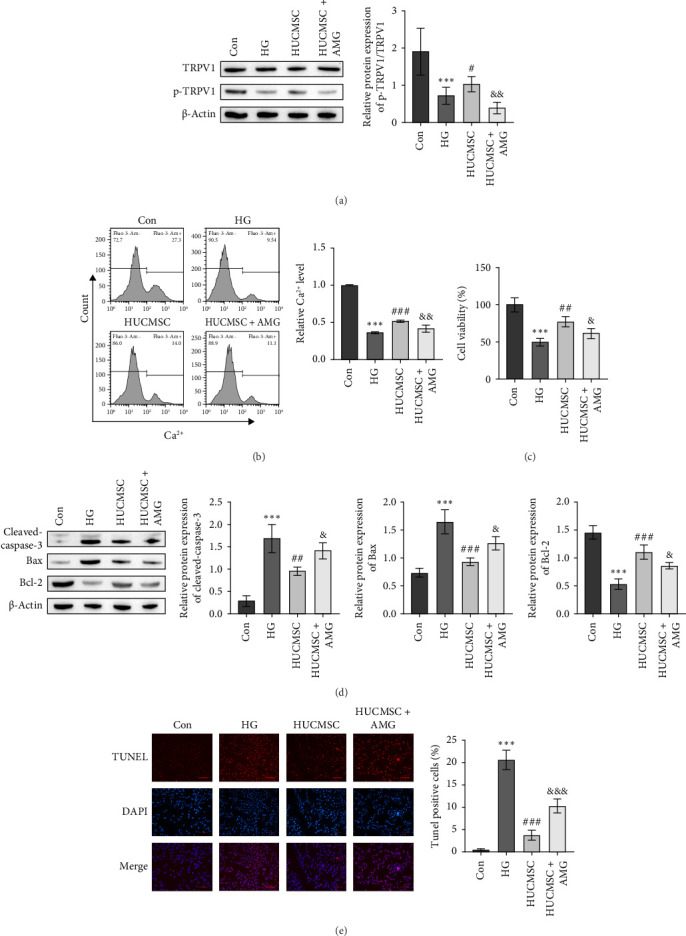
Umbilical cord mesenchymal stem cells alleviate high glucose-induced Schwann cell injury through the Ca^2+^ signaling pathway. (A) Western blot was used to detect the expression of p-TRPV1 and TRPV1. (B) Flow cytometry was employed to detect the Ca^2+^ level. (C) The CCK-8 assay was utilized to detect cell proliferation viability. (D) Western blot was used to detect the expression of apoptosis-related proteins Bax, Bcl-2, and cleaved-caspase-3. (E) Tunel staining was performed to detect cell apoptosis. Compared with the Con group, *⁣*^*∗∗∗*^*p* < 0.001; compared with the HG group, ^#^*p* < 0.05, ^##^*p* < 0.01, ^###^*p* < 0.001; and compared with the HUCMSC group, ^&^*p* < 0.05, ^&&^*p* < 0.01, ^&&&^*p* < 0.001. AMG: the TRPV1 antagonist, AMG9810 (*n* = 3; full-length blots are presented in Figure S2A,D).

To investigate the molecular mechanisms by which HUCMSCs improve diabetic neuropathy, we cocultured mouse SCs under HG conditions with HUCMSCs and analyzed the role of the TRPV1 signaling pathway in this process. Western blot analysis showed that HG significantly decreased the p-TRPV1/TRPV1 ratio in mouse SCs, whereas coculture with HUCMSCs increased this ratio. However, the addition of AMG9810 reduced the elevated p-TRPV1/TRPV1 levels induced by HUCMSCs (Figure 2A). Subsequently, flow cytometry revealed that HG significantly lowered intracellular Ca^2+^ levels in mouse SCs, which were markedly increased after coculture with HUCMSCs but decreased again upon addition of AMG9810 (Figure 2B). CCK-8 assays demonstrated that HG significantly inhibited the proliferation of mouse SCs, which was enhanced by coculture with HUCMSCs but attenuated by the addition of AMG9810 (Figure 2C). Western blot analysis of apoptosis-related proteins, cleaved-caspase-3, Bax, and Bcl-2 in mouse SCs showed that HG increased the expression of cleaved-caspase-3 and Bax while decreasing Bcl-2 expression. Coculture with HUCMSCs significantly decreased the expression of cleaved-caspase-3 and Bax while increasing Bcl-2 expression. These effects were reversed by the addition of AMG9810 (Figure 2D). TUNEL staining indicated that HG promoted apoptosis in mouse SCs, which was significantly reduced by coculture with HUCMSCs but increased again when AMG9810 was added during coculture (Figure 2E). These results suggest that HUCMSCs alleviate HG-induced SC injury by promoting the TRPV1-mediated Ca^2+^ signaling pathway, reducing apoptosis, and enhancing cell proliferation.

### 3.3. HUCMSCs Regulate the AMPK Signaling Pathway via TRPV1

**Figure 3:**
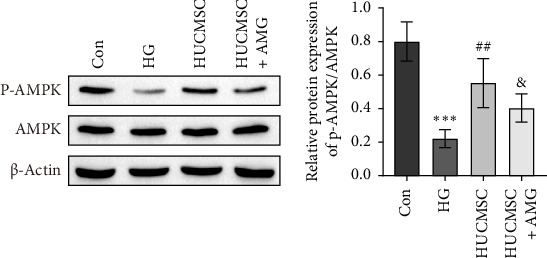
Human umbilical cord mesenchymal stem cells alleviate high glucose-induced Schwann cell injury by regulating AMPK through TRPV1. The expression of p-AMPK and AMPK was detected by Western blot. Compared with the Con group, *⁣*^*∗∗∗*^*p* < 0.001; compared with the HG group, ^##^*p* < 0.01; and compared with the HUCMSC group, ^&^*p* < 0.05. AMG: The TRPV1 antagonist AMG9810 (*n* = 3; full-length blots are presented in Figure S3).

To further verify the specific mechanisms by which HUCMSCs influence SCs under HG conditions via TRPV1, we examined the relationship between TRPV1 and AMPK. Western blot analysis showed that HG significantly decreased the p-AMPK/AMPK ratio, whereas coculture with HUCMSCs markedly increased this ratio. However, the addition of AMG9810 (a TRPV1 antagonist) significantly reduced the p-AMPK/AMPK ratio (Figure 3). These results indicate that HUCMSCs exert a protective effect by activating the AMPK signaling pathway through TRPV1.

### 3.4. HUCMSCs Alleviate HG-Induced SC Mitochondrial Dysfunction via the AMPK Signaling Pathway

**Figure 4:**
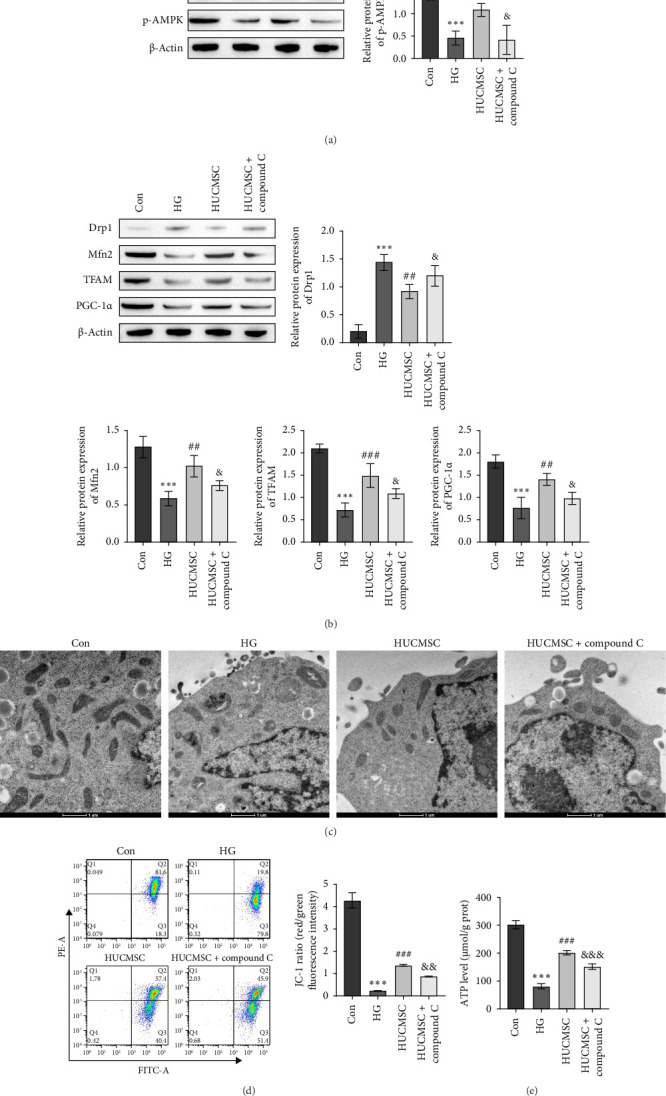
Umbilical cord mesenchymal stem cells alleviate high glucose-induced mitochondrial dysfunction in Schwann cells via the AMPK signaling pathway. (A) Western blot was used to detect the expression of p-AMPK and AMPK. (B) Western blot was used to detect the expression of Drp1, Mfn2, TFAM, and PGC-1α. (C) Mitochondrial morphology changes were observed using an electron microscope. (D) Flow cytometry for detecting mitochondrial membrane potential. (E) The ATP level was evaluated using a kit. Compared with the Con group, *⁣*^*∗∗∗*^*p* < 0.001; compared with the HG group, ^#^*p* < 0.05, ^##^*p* < 0.01, ^###^*p* < 0.001; and compared with the HUCMSC group, ^&^*p* < 0.05. AMPK inhibitor: compound C (*n* = 3; full-length blots are presented in Figure S4A,B).

Having established that HUCMSCs improve HG-induced SC injury through TRPV1-mediated Ca^2+^ and AMPK signaling pathways, we further investigated the underlying mechanisms by treating mouse SCs with the AMPK inhibitor compound C and assessing downstream markers. Western blot analysis revealed that HG significantly decreased the p-AMPK/AMPK ratio, which was markedly increased following coculture with HUCMSCs but significantly reduced by the addition of compound C (Figure 4A). Additionally, Western blot analysis showed that HG significantly increased the expression of the mitochondrial function-related protein Drp1 and decreased the expression of PGC-1α, TFAM, and Mfn2 in mouse SCs. Coculture with HUCMSCs reduced Drp1 expression and increased the expression of PGC-1α, TFAM, and Mfn2, but these effects were reversed by the addition of compound C (Figure 4B). Furthermore, the morphological changes of mitochondria in SCs were observed using a transmission electron microscope. HG treatment led to mitochondrial fragmentation and mitochondrial cristae loss. HUCMSCs attenuated the HG-induced mitochondrial fragmentation. However, the addition of compound C weakened the protective effect of HUCMSCs (Figure 4C). Further, flow cytometry and ATP kits were used to detect mitochondrial membrane potential and ATP levels. The results showed that HG significantly reduced mitochondrial membrane potential and ATP levels. These levels increased after coculture with HUCMSCs. Further compound C treatment decreased mitochondrial membrane potential and ATP levels (Figure 4D,E). These findings suggest that HUCMSCs alleviate HG-induced SC injury by improving mitochondrial function through AMPK signaling.

### 3.5. Schematic Illustration of the Mechanism

HUCMSCs activate the TRPV1-[Ca^2+^]i-AMPK signaling pathway. They regulate the mitochondrial biogenesis of SCs through PGC-1α and TFAM and control mitochondrial fission and fusion via Drp1 and Mfn2. This, in turn, weakens the proapoptotic effect of HG on SCs.


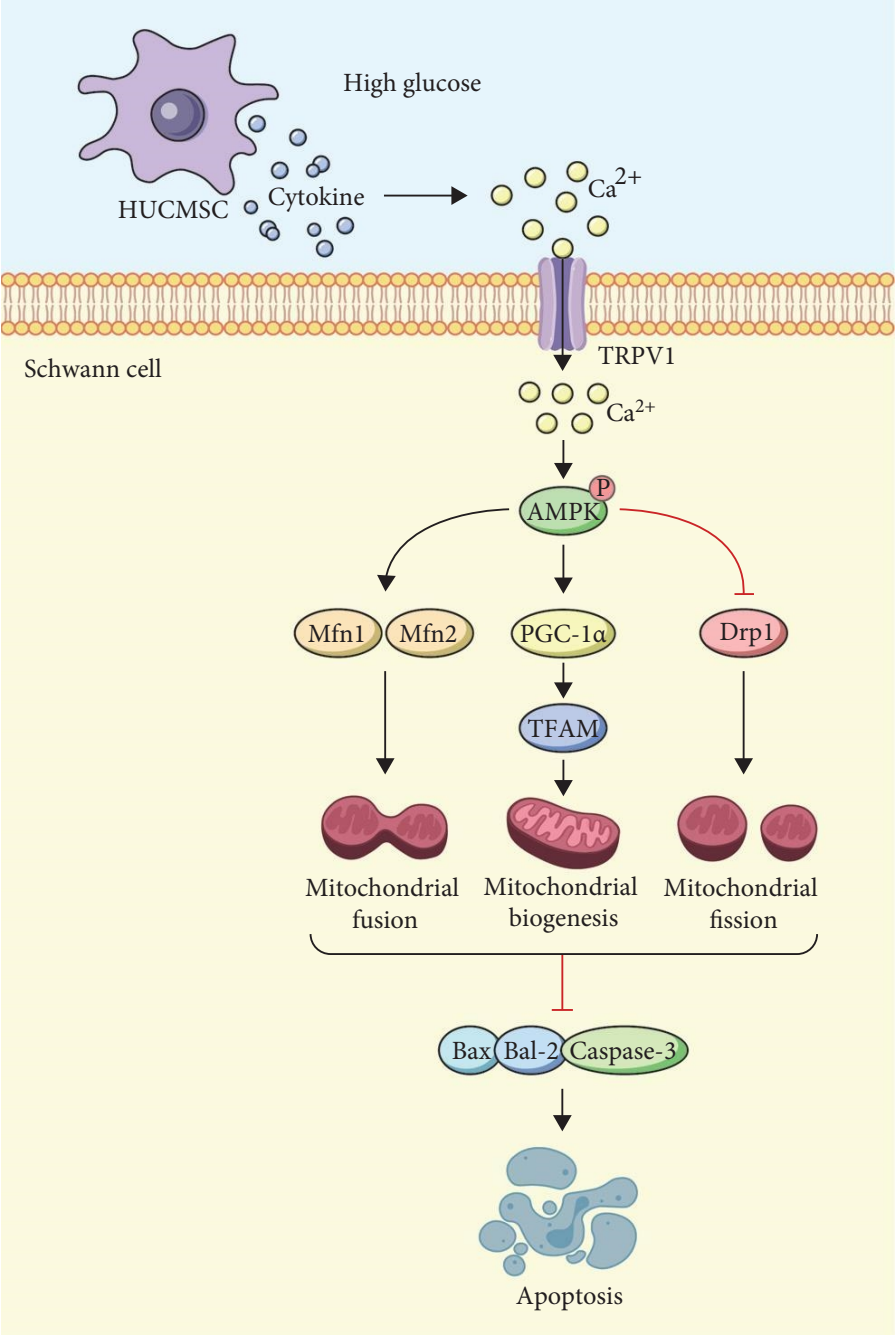


## 4. Discussion

This study demonstrates that HUCMSCs effectively alleviate mechanical allodynia and thermal hyperalgesia in DPN mice. Mechanistically, we identify a previously unrecognized TRPV1-Ca^2+^-AMPK signaling axis in SCs as the core pathway mediating HUCMSC therapy. HG suppressed the p-TRPV1/TRPV1 ratio and intracellular Ca^2+^ influx in SCs, concomitant with reduced proliferation and increased apoptosis. Strikingly, HUCMSC treatment reversed these deficits by enhancing TRPV1 phosphorylation and Ca^2+^ influx, thereby reducing apoptosis, revealing SC TRPV1 as a novel therapeutic target for DPN.

In painful DPN models, pain phenotypes vary across studies, with reports of biphasic thermal responses [13, 28] or concurrent mechanical/thermal hypersensitivity [15, 16]. Our DPN model exhibited reduced mechanical sensitivity but heightened thermal hyperalgesia, suggesting model-specific neurofunctional adaptations. Critically, HUCMSCs normalized both sensory modalities, underscoring their broad therapeutic potential regardless of pain phenotype heterogeneity.

TRPV1, a calcium-permeable nonselective cation channel, integrates diverse pain stimuli [29–32] and is implicated in DPN pathogenesis [33]. While diabetic rodent models show conflicting TRPV1 expression changes in dorsal root ganglia (DRG) [15, 16, 18, 28, 34], our study is the first to report glucose-dependent TRPV1 dysregulation specifically in SCs, a type of glial cell pivotal for axonal integrity yet understudied in DPN mechanisms. TRPV1 activation exerts protective effects in diabetes, including mitigating oxidative stress in DRG [18] and preserving vascular function [19]. Here, we extend these benefits to SCs by demonstrating that HUCMSC-enhanced TRPV1 phosphorylation directly counteracts HG-induced apoptosis, providing a new cytoprotective strategy.

We further delineated the downstream signaling cascade: HUCMSCs upregulated the p-AMPK/AMPK ratio via TRPV1 activation, positioning AMPK as a key effector of TRPV1 in SCs. This interaction aligns with TRPV1-AMPK crosstalk observed in other systems [35, 36]. Notably, HG concurrently inhibits TRPV1 [20] and AMPK phosphorylation [20, 21], suggesting their coordinated dysfunction drives DPN progression. Our discovery that HUCMSCs rescue both pathways offers a unified mechanistic framework for their efficacy. Furthermore, our research has demonstrated that HUCMSCs can exert protective effects via p-AMPK upregulation. Some studies have also shown that metformin can activate AMPK and improve DPN [37]. However, long-term use of metformin can increase the risk of DNP [38, 39]. The HUCMSCs therapy addresses the root cause of the disease by promoting nerve regeneration and recovery. Unlike drug interventions that merely control neurological symptoms, this approach has the potential to slow down or even reverse the progression of the disease [3]. Therefore, HUCMSCs therapy has a higher safety.

Central to this mechanism is TRPV1-mediated Ca^2+^ influx. Although diabetes alters TRPV1 currents in DRG neurons [13, 14, 28, 33, 34, 40, 41], SC Ca^2+^ dynamics remain largely unexplored. We found HG suppressed Ca^2+^ influx in SCs, whereas HUCMSCs restored it via TRPV1, an effect abolished by TRPV1 antagonism. This SC-specific Ca^2+^ regulation represents a significant advance in understanding DPN pathophysiology. TRPV1-driven Ca^2+^ flux activates AMPK through calcium/calmodulin-dependent protein kinase kinase 2 (CAMKK2) [42–46], a pathway validated in vascular endothelial cells [20] and renal podocytes [24]. Our work establishes its operational relevance in SCs, with HUCMSCs potentiating the TRPV1/[Ca^2+^]i/AMPK axis to combat glucose toxicity.

Downstream of AMPK, we uncovered HUCMSC-mediated restoration of mitochondrial homeostasis, a critical innovation given mitochondrial dysfunction is an important cause of DPN [47–50]. The mitochondrial biogenesis key protein PGC-1α, mitochondrial DNA replication/transcription key proteins TFAM, mitochondrial fusion protein Mfn2, and mitochondrial fission protein Drp1 play significant roles in maintaining mitochondrial function. In the HG environment, mitochondrial biosynthesis (regulated by PGC-1α/TFAM) and fusion (mediated by Mfn2) are reduced, while mitochondrial fission (mediated by Drp1) is increased [51]. Our research in SCs also yielded consistent results, and HUCMSCs can weaken the effect of HG and partially restore the mitochondrial function in SCs. AMPK orchestrates mitochondrial biogenesis via PGC-1α/TFAM signaling [52–56], which is impaired in diabetic tissues [51, 57]. Here, HUCMSCs reversed glucose-induced PGC-1α/TFAM suppression in an AMPK-dependent manner, directly linking TRPV1 activation to mitochondrial biogenesis in SCs for the first time.

Equally important, HUCMSCs rebalanced mitochondrial dynamics by modulating fission/fusion proteins. HG elevated the fission protein Drp1 while reducing the fusion protein Mfn2, changes associated with apoptosis [25–27] and diabetic complications [58, 59]. HUCMSCs normalized both Drp1 and Mfn2 expression, thereby inhibiting excessive fission. Although AMPK typically promotes fission via Drp1 phosphorylation [60], our findings suggest HUCMSCs may override this by enhancing fusion (via Mfn2 upregulation) to achieve net mitochondrial stabilization, a nuanced regulatory mechanism with therapeutic implications. AMPK physically interacts with Mfn2 [61], and Mfn2 deficiency exacerbates diabetic injury [62–64]. Furthermore, this study further demonstrated that HG reduced mitochondrial membrane potential and ATP levels, exacerbating mitochondrial dysfunction. However, HUCMSCs alleviated this process. Thus, HUCMSC-driven AMPK activation likely coordinates both biogenesis and dynamics to restore mitochondrial integrity.

Although our research has achieved encouraging results, the use of HUCMSCs as a clinical therapy for DPN still faces challenges. Firstly, the standardization of the isolation and amplification protocols and characterization methods of HUCMSCs remains a key issue to ensure reproducibility and effectiveness in different research and clinical settings [65]. Secondly, the treatment with donor-derived HUCMSCs has potential risks of immune rejection, and their interaction with the microenvironment brings uncertainties to their clinical application [66, 67]. Furthermore, the progress of DPN at different stages may vary among patients. Optimizing the delivery method and adjusting the dosage to enhance the implantation, survival, and targeted tissue localization of HUCMSCs remains a major challenge [3]. Finally, continuous long-term monitoring is crucial to assess the safety of HUCMSCs treatment. Therefore, more research is needed in the future to explore the role of HUCMSCs therapy and the HUCMSCs-mediated TRPV1-[Ca^2+^]i-AMPK signaling axis in alleviating DPN.

## 5. Conclusion

This study establishes HUCMSCs as multitarget therapies for DPN by activating the TRPV1-Ca^2+^-AMPK axis in SCs, thereby rescuing mitochondrial biogenesis (via PGC-1α/TFAM) and dynamics (via Drp1/Mfn2) to suppress apoptosis (Graphical Abstract). In vivo efficacy in ameliorating diabetic neuropathic pain was abrogated by TRPV1 or AMPK inhibition, clinically validating this pathway's therapeutic relevance. Our work significantly advances DPN treatment paradigms by (1) identifying SC TRPV1 as a glucose-sensing target, (2) defining mitochondrial restoration as a primary mechanism of HUCMSCs, and (3) proposing TRPV1 agonists as promising nonopioid alternatives for pain management. These insights accelerate translational applications of HUCMSC-based therapies and TRPV1 modulators for diabetic neuropathy.

## Data Availability

All additional files are included in the manuscript.

## Supporting Information

Supporting Information The full uncropped Gels and Blots images related to Figures 2–4.
