# Supplementary material for: Human Umbilical Cord Mesenchymal Stem Cells Ameliorate Diabetic Neuropathic Pain via TRPV1-[Ca2+]i-AMPK Signaling-Mediated Mitochondrial Restoration in Schwann Cells
Source: Stem Cells Int. 2025 Nov 26;2025:5533136. doi: 10.1155/sci/5533136 (PMC12674885; doi:10.1155/sci/5533136)

Figure 2A

TRPV1(75KDa)


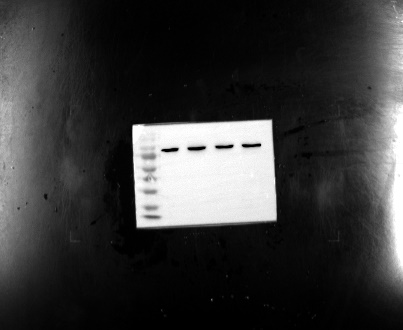

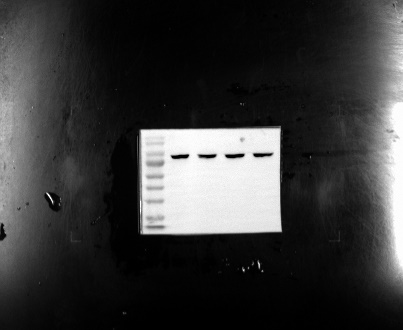
Repeat 1 Repeat 2 Repeat 3


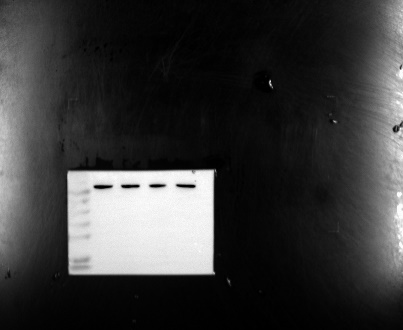


p-TRPV1(75KDa)

Repeat 1 Repeat 2 Repeat 3


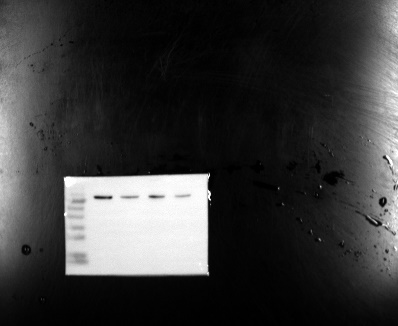

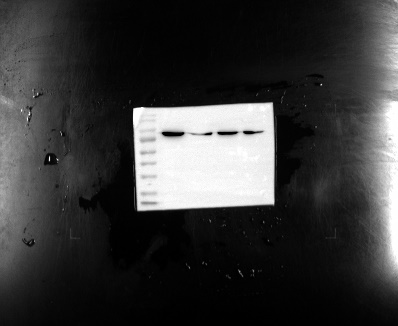

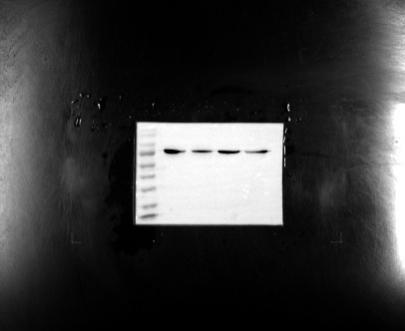


β-actin(42KDa)

Repeat 1 Repeat 2 Repeat 3


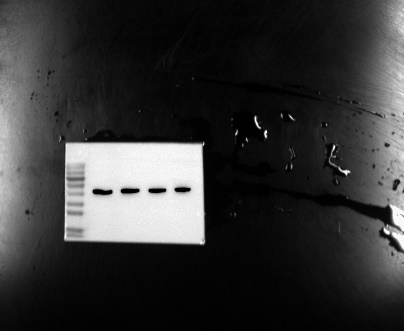

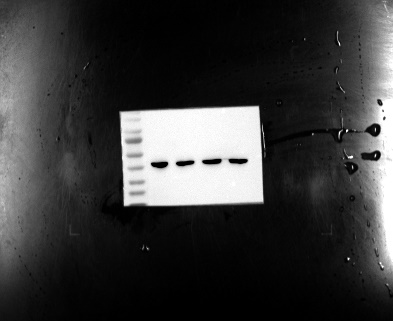

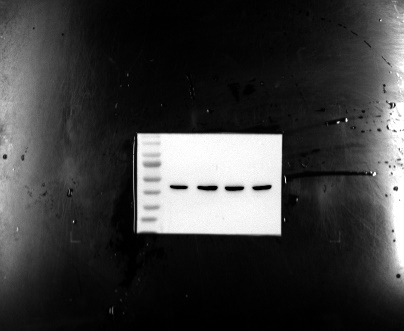


Figure 2D

Cleaved-caspase-3(32KDa)

Repeat 1 Repeat 2 Repeat 3


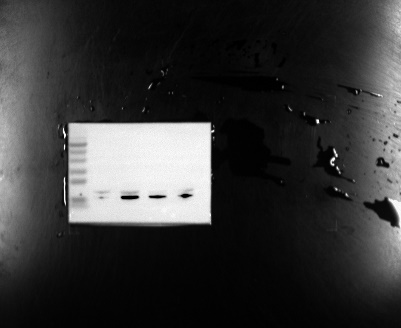

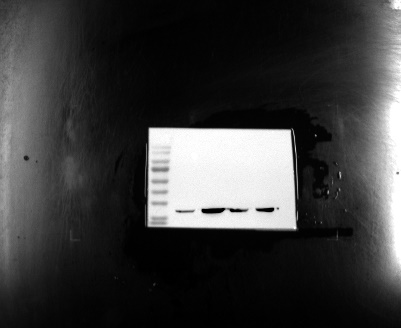

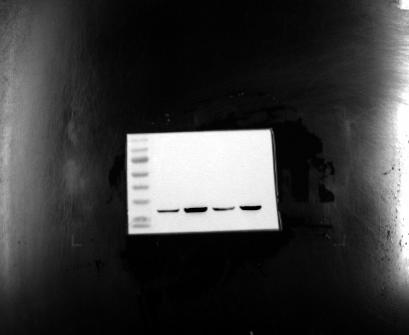


Bax(20KDa)

Repeat 1 Repeat 2 Repeat 3


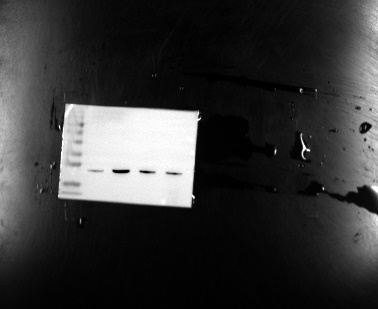

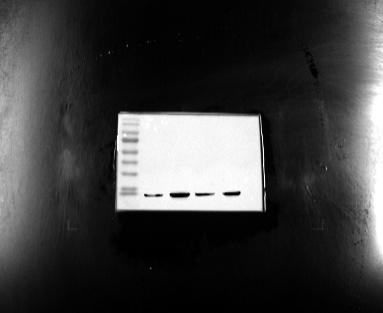

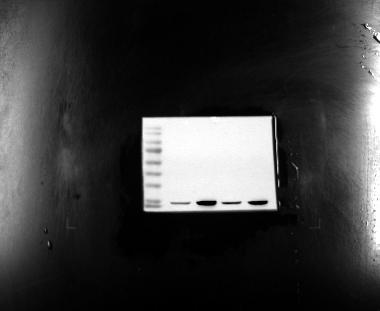


Bcl-2(26KDa)

Repeat 1 Repeat 2 Repeat 3


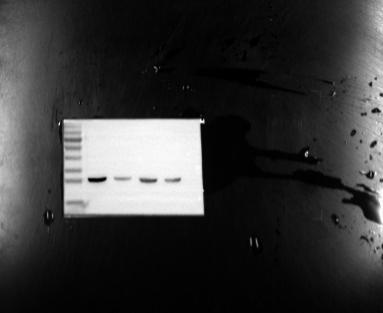

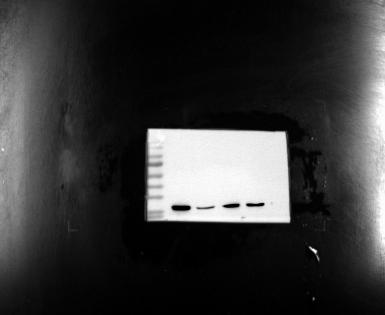

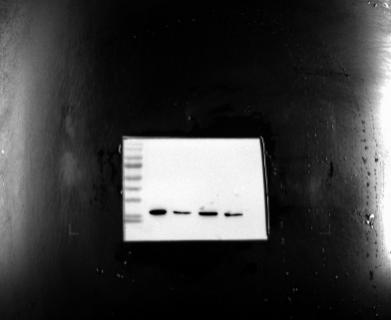


β-actin(42KDa)

Repeat 1 Repeat 2 Repeat 3


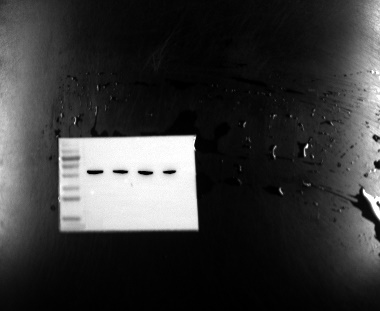

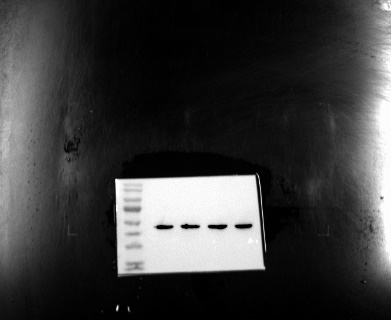

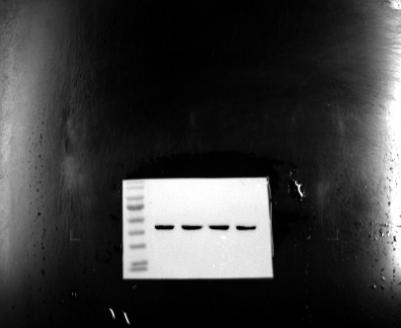


Figure 3A

AMPK(64KDa)

Repeat 1 Repeat 2 Repeat 3


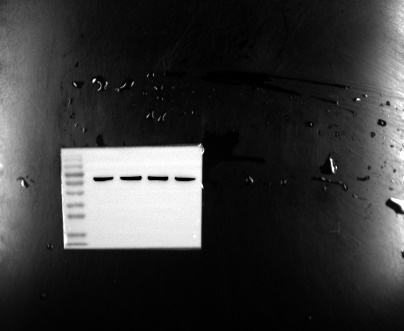

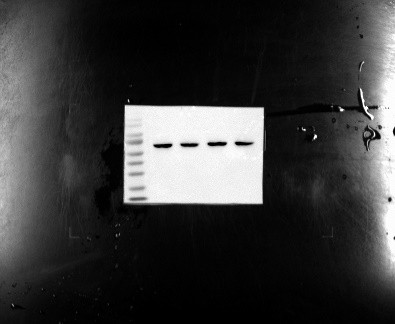

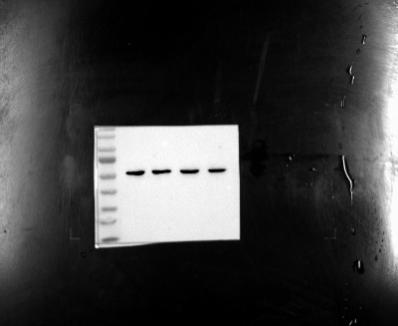


p- AMPK (64KDa)

Repeat 1 Repeat 2 Repeat 3


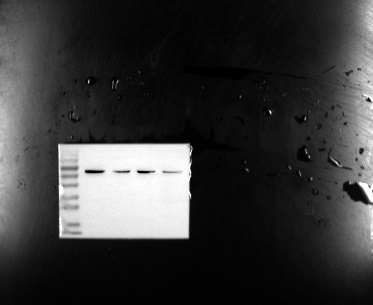

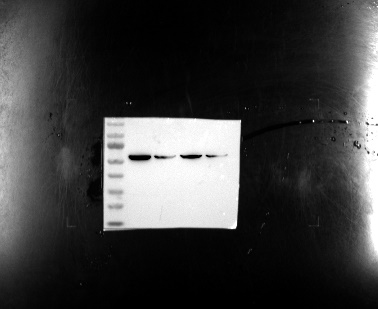

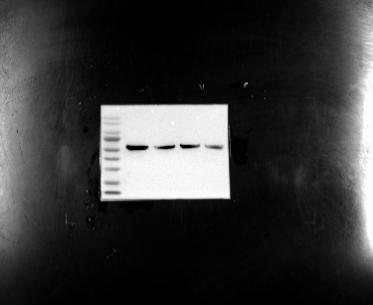


β-actin(42KDa)

Repeat 1 Repeat 2 Repeat 3


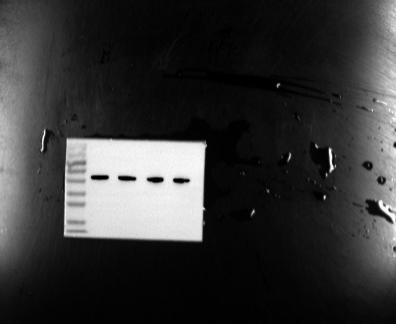

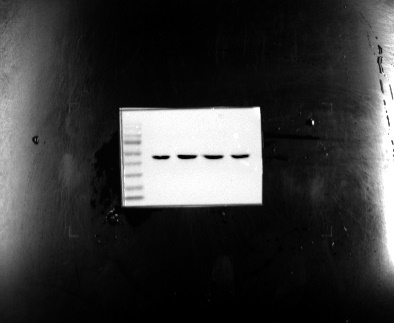

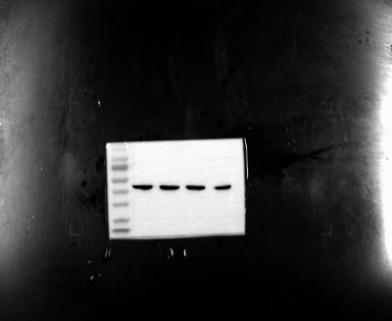


Figure 3B

Drp1(83KDa)

Repeat 1 Repeat 2 Repeat 3


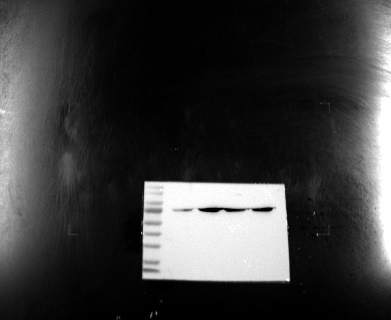

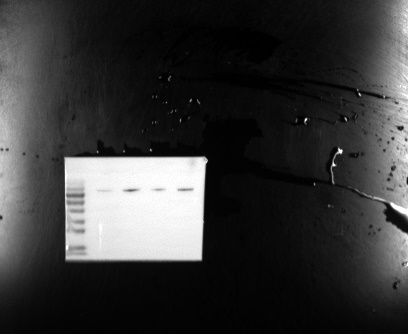

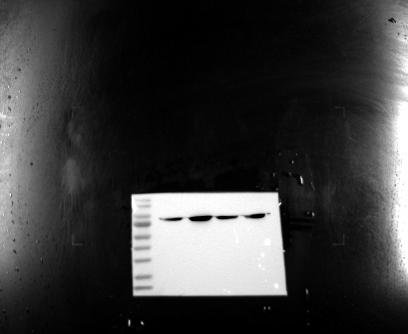


Mfn2(80KDa)

Repeat 1 Repeat 2 Repeat 3


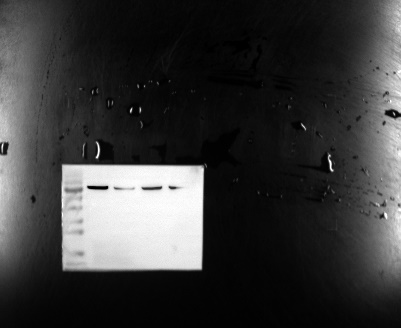

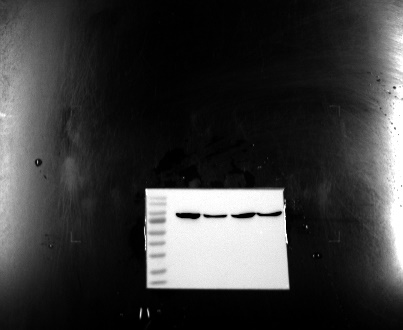

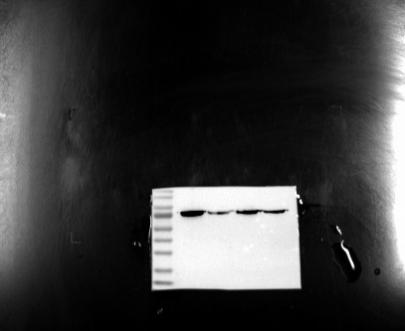


TFAM(25KDa)

Repeat 1 Repeat 2 Repeat 3


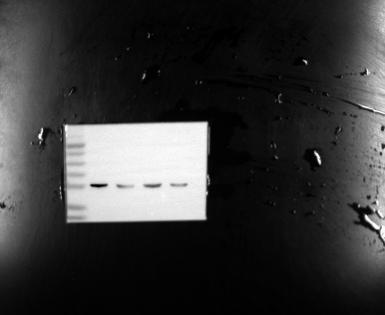

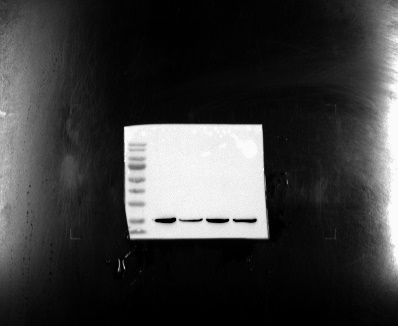

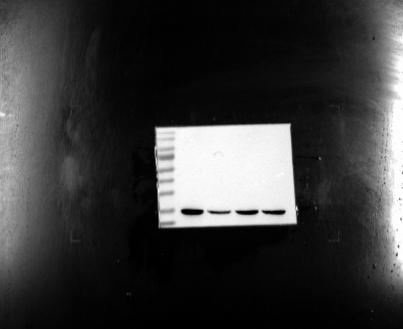


PGC-1α(98KDa)

Repeat 1 Repeat 2 Repeat 3


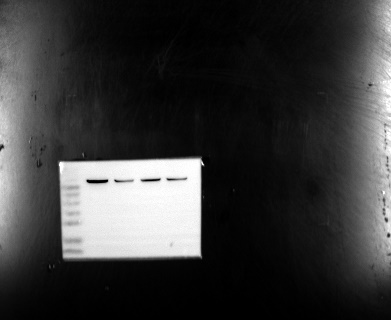

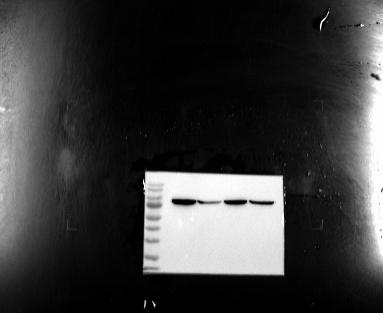

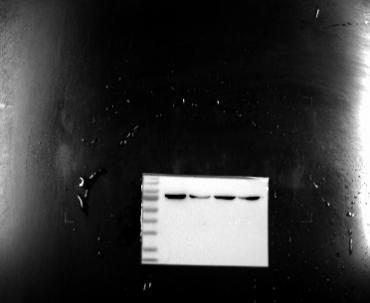


β-actin(42KDa)

Repeat 1 Repeat 2 Repeat 3


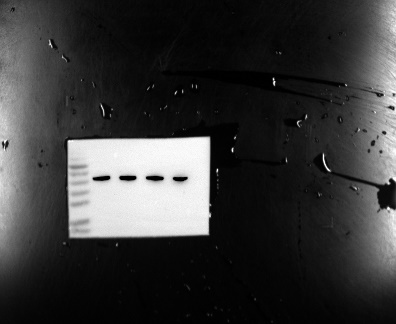

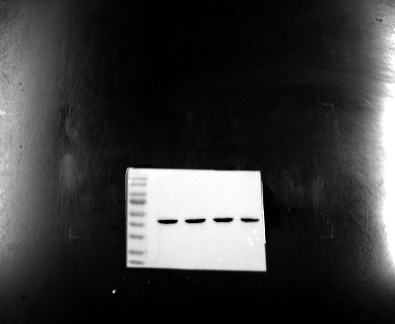

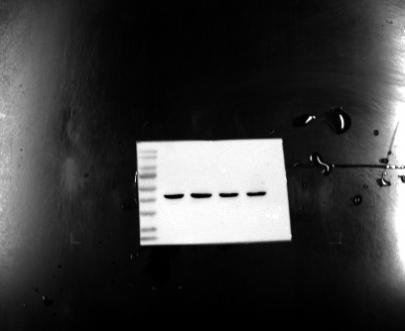


Figure 4

AMPK(64KDa)

Repeat 1 Repeat 2 Repeat 3


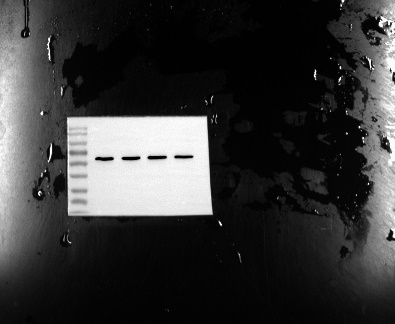

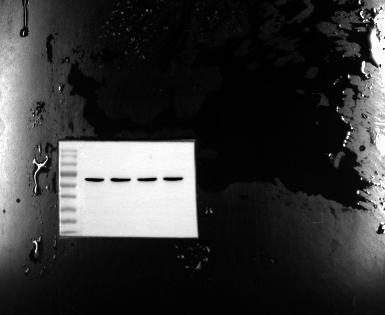

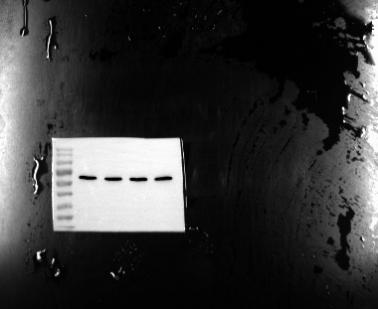


p- AMPK (64KDa)

Repeat 1 Repeat 2 Repeat 3


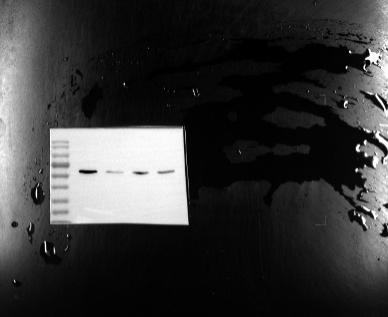

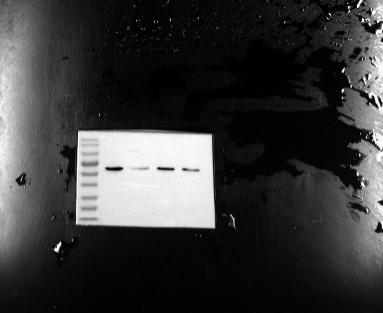

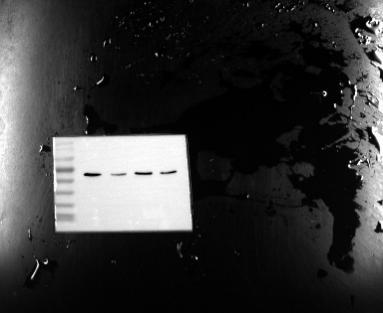


β-actin(42KDa)

Repeat 1 Repeat 2 Repeat 3


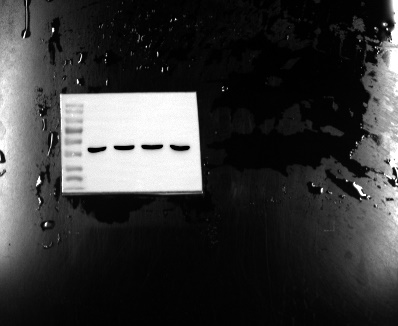

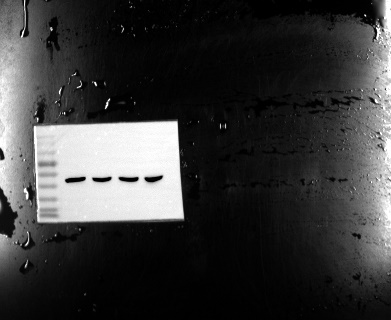

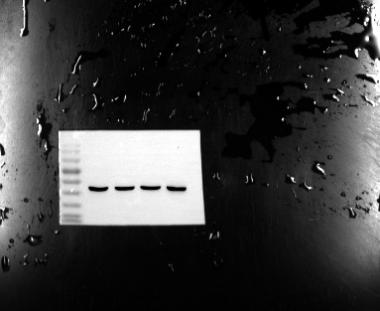

Supplement: Supporting Information — The full uncropped Gels and Blots images related to Figures 2–4. [file 5533136.f1.docx]
